# Editorial: Current perspectives in theory and research on the role of gender in environmental psychology

**DOI:** 10.3389/fpsyg.2026.1917460

**Published:** 2026-07-29

**Authors:** Lorenza Tiberio, Louise Eriksson, Anna Bornioli

**Affiliations:** 1 Department of Education, Roma Tre University, Rome, Italy; 2 Department of Geography, Umeå University, Umeå, Sweden; 3 SGlobal, Barcelona Institute for Global Health, Barcelona, Spain

**Keywords:** environmental psychology, gender, intersectionality, pro-environmental behavior, social inclusion

This Research Topic brings together contributions that illustrate gender influence on attitudes, perceptions, and behaviors related to the environment. Collectively, these studies demonstrate that environmental experiences are shaped by gendered norms, identities, cultural expectations, and structural inequalities rather than being gender-neutral (Brough et al., 2016). Overall, the studies contained in this Research Topic contribute to the growing body of research which challenges universalistic assumptions in environmental psychology and emphasizes the importance of more inclusive and culturally sensitive perspectives on the human–environment relationships. The perspective is consistent with recent research regarding people's interaction with the environment, which demonstrates that regenerative experiences in urban contexts are not socially neutral, but can differ depending on the gender, both in preferred indoor and outdoor locations as well as in urban green spaces (Subiza-Pérez et al., 2021; Bornioli et al., 2024).

Two complementary lines of inquiry can be discerned from the four contributions included in this Research Topic. The first line of inquiry concerns the relationship between gender identity and pro-environmental behaviors. Chard et al. examine sustainable diet intentions in the United Kingdom, China, Sweden, and Brazil, demonstrating that gender differences in sustainable diet intentions vary by cultural context. Their findings indicate that dietary identity, subjective norms, environmental concerns, and the perceived social status of meat consumption are associated with sustainable diet intentions differently depending on gender and country of origin. Such results illustrate that gender differences are not universal but rather culturally determined, suggesting that sustainable eating practices are closely related to culturally constructed meanings of masculinity and femininity. This study emphasizes the importance of adopting a transnational research design beyond predominantly Western frameworks to more fully understand gendered practices. Remsö et al. examine climate change denial in Sweden through the concept of threatened masculinity to explore questions related to masculinity and environmental commitment. According to their findings, men are more likely than women to deny climate change, and this tendency can be explained in part by the perception that changing gender relations threaten masculinity. The authors hypothesize that denying climate change may function as a symbolic reaffirmation of traditional masculinity in instances where environmental concerns are culturally associated with femininity. This contribution demonstrates the importance of integrating environmental psychology, political psychology, and gender studies to gain insight into the emotional and identity dimensions of resistance to climate action.

The second line of inquiry concerns gendered experiences of urban and environmental spaces. Su et al. describe how men and women experience urban spaces differently during nighttime physical activity. In their qualitative research, the authors identify both physical environmental factors as well as environmental perceptions as relevant for the participation of people in nighttime physical activities. Based on their findings, women place a greater emphasis on safety, comfort, emotional experience, and a sense of social belonging than men do, whereas men pay more attention to functional and performance factors. A key element of the study is the importance of taking gender perspectives into account in urban planning and environmental design, especially in regard to wellbeing, accessibility, and perceptions of safety. Generally, the paper reinforces the notion that environmental spaces are not only physically occupied but are also socially experienced and emotionally interpreted.

These empirical findings have conceptual implications, which are explicitly addressed by Pinho, who calls for the development of an environmental psychology that is more inclusive and intersectional. The purpose of this theoretical and prospective article is to provide a critical examination of the relationship between environmental psychology, gender, and social inclusion. In her essay, the author argues that traditional environmental psychology have historically used models that are perceived as gender-neutral, but reflect dominant perspectives and experiences, often those belonging to western cultures and men. A particular focus is placed on intersectionality and the need to consider the experiences of women, marginalized groups, and communities at risk because of the disproportional effects of environmental risks faced by these groups. As a conclusion, the article proposes an environment psychology approach that is more critical, participatory, and socially just, capable of incorporating feminist and intersectional approaches into research, but also to contribute to systemic transformation in practice in fields such as urban planning and environmental governance.

## Conclusions and future directions

This Research Topic is intended to provide a starting point for identifying promising directions for future interdisciplinary research, rather than a comprehensive overview of gendered processes in environmental psychology. The contributions support the need to examine gender in environmental psychology using different theoretical models and methodological approaches, with three findings standing out. First, the studies provide empirical support for how identity processes, social norms, and culturally constructed gender roles contribute to the formation of environmental attitudes and behaviors. Second, gender emerges as a key factor for environmental perceptions developed not only based on the physical context itself, but also emotional, symbolic, and relational dimensions. Third, taking a more comprehensive view of gender and intersectionality can greatly enhance theoretical understandings in environmental psychology. Jointly, the contributions have implications for practical interventions in fields such as climate communication, environmental protection, and urban planning. As a final note, this Research Topic contributes to ongoing efforts to broaden the scope of environmental psychology and to develop more inclusive and socially-based approaches to environmental research. A gender-sensitive and intersectional perspective is essential at a time when environmental crises intersect with social inequalities to better understand the complex relationship between humans and the environment. Although these contributions emphasize sustainable behavior, climate attitudes, urban experiences, as well as theoretical developments, other important topics remain underrepresented, including considerations of how gender and psychological factors interact in natural resource management, longitudinal research on gendered environmental experiences across the life course, studies conducted in low- and middle-income countries, and work examining how gender intersects with other dimensions of inequality, including age, disability, ethnicity, socioeconomic status, and sexual identity. Similarly, more consideration could be given to interventions that evaluate the impact of gender-sensitive environmental policies and urban design on wellbeing and pro-environmental behavior in everyday settings.
